# The evolving landscape of African swine fever: the game-changing impact of recombinant genotype I/II on Asia–Pacific control strategies

**DOI:** 10.3389/fvets.2026.1855026

**Published:** 2026-07-15

**Authors:** Jesper Chia-Hui Hsu, Rachel A. Schambow, Yu-Liang Huang, Vo Dinh Chuong, John M. Humphreys, Andres M. Perez

**Affiliations:** 1 Center for Animal Health and Food Safety, College of Veterinary Medicine, University of Minnesota, Minneapolis, MN, United States; 2 Veterinary Research Institute, Ministry of Agriculture, Executive Yuan, New Taipei City, Taiwan; 3 Vietnam Department of Animal Health and Production, Hanoi, Vietnam; 4 U.S. Department of Agriculture, Agricultural Research Service, National Bio and Agro-Defense Facility, Manhattan, KS, United States

**Keywords:** African swine fever virus, diagnostic, genotype I, p72 genotype II, recombinant strains

## Abstract

The emergence of genotype I/II recombinant African swine fever virus (rASFV) in the Asia–Pacific region represents a significant shift in the molecular epidemiology of the ongoing ASF panzootic. Following the PRISMA-ScR framework, this study synthesizes sources of evidence from China, Vietnam, Russia, and Taiwan published between 2021 and 2025. Four electronic databases and relevant gray literature were included in the review. The evidence indicates that “JS/LG/21-like” recombinant strains exhibit a stable chimeric genome architecture, comprising a genotype I backbone incorporating genotype II-associated virulence determinants, including *EP402R* and members of the *MGF_505/360* gene families. This genomic mosaicism appears to confer a synergistic phenotype characterized by high lethality and preserved hemadsorption (HAD+) activity, exceeding the typically lower virulence profile of ASF genotype I lineages. The research further identifies key systemic vulnerabilities. First, diagnostic platforms relying primarily on genotype II-specific primers may lead to misclassification or under-detection of recombinant strains. Second, existing genotype II-targeted vaccine candidates exhibit limited cross-protective immunity against rASFV. Comparative genomic analyses demonstrate extremely high nucleotide identity (>99.9%) across affected regions, supporting the hypothesis of a monophyletic evolutionary profile followed by rapid clonal expansion. These findings highlight the urgent need to update diagnostic platforms, expand high-resolution genomic surveillance (e.g., whole genome sequencing), and integrate sylvatic cycle dynamics into routine monitoring frameworks. Strengthened policy coordination and One Health-oriented strategies are essential to mitigate the sustained regional transmission and ecological entrenchment of these stabilized recombinant lineages.

## Introduction

African swine fever (ASF) is a highly contagious viral disease with high mortality rates in acute cases, affecting both domestic pigs and wild boar (1). ASF is caused by a large double-stranded DNA virus in the *Asfarviridae* family generically referred to as ASF virus (ASFV) (2). Characterized by an icosahedral capsid containing the major p72 structural protein and a genome of 170–190 kbp. ASFV has been classified into 24 genotypes based on *B646L* gene. Due to its transboundary nature and devastating impact on international trade and economic stability, ASF is designated as a notifiable disease by the World Organization for Animal Health (WOAH). While it has been endemic to sub-Saharan Africa since its first description in East Africa in 1921, the global epidemiological landscape shifted dramatically in the late 2010s, significantly impacting the Asia–Pacific regions (3).

The introduction of genotype II ASF into the northeastern city of Shenyang, China in 2018 marked the beginning of an unprecedented regional crisis and a new animal disease pandemic (4). Between 2019 and late 2025, the virus surged across the Asia–Pacific, with more than 20 countries reporting outbreaks in either domestic swine or wild boar populations. The socio-economic consequences have been catastrophic for most countries. Asia produces over half of the world's pork and raises 74% of the global pig population (5, 6). The impact of the ASF epidemic has been particularly severe in major production hubs. According to official statistics, China—the world's largest swine producer—experienced an approximate 40% reduction in its national pig inventory between 2018 and 2019 (7), followed by a 30-month cycle of decline and recovery (8). Similarly, Vietnam, the largest swine-producing country in Southeast Asia, has seen the outbreak spread across all 63 provinces and municipalities in less than 1 year after its introduction in February 2019, leading to the death or culling of more than 6 million pigs in the initial epidemic year. Such reduction represents over 20% of its national herd, severely destabilizing the agricultural sector and rural livelihoods (9). In the Philippines, ASF outbreaks have now reached all 17 administrative regions and 76 of the 82 provinces, leading to a total loss of 5 million pigs through depopulation efforts (10). Most recently, in October 2025, Taiwan recorded its first domestic pig case, prompting an aggressive response that included a 15-day movement ban. Despite earmarking $35 million USD for prevention, the comprehensive economic toll of this localized outbreak reached $67 million USD due to supply chain disruption and subsequent subsidies (11).

ASF containmentin Asia–Pacific is challenged by a fragmented production system. According to the Food and Agriculture Organization of the United Nations (FAO) reports, roughly 80% of pig output in Asia relies on mixed crop-livestock systems, which are predominantly smallholder farms, or backyard farming (12). Although the definition of a “smallholder” varies by region—for example, under 20 head of pigs in the Philippines vs. 1–10 head in Vietnam—these non-commercial production systems typically operate with limited biosecurity. Practices such as swill feeding remain common, increasing the risk of disease introduction and transmission (13). As a result, these systems are particularly vulnerable to transboundary animal diseases, including foot and mouth disease (FMD), classical swine fever (CSF) and ASF (14–16). Despite governmental interventions responding nearly immediately after the virus's introduction—including movement controls, zoning (e.g., the Philippines), and compensation schemes—and the 2023 release of Vietnamese vaccines (NAVET-ASFVAC and AVAC ASF LIVE), ASF remains poorly controlled (17, 18). The disease is now increasingly endemic, with recurrence linked to seasonal factors and human movement.

In June 2021, non-haemadsorbing genotype I ASFVs were first reported on pig farms in Shandong (SD/DY-I/21) and Henan (HeN/ZZ-P1/21) provinces in China (19). Subsequently, recombinant genotype I/II ASFV strains were detected in Jiangsu Province in 2021, representing a significant shift in the ASF epidemiological landscape. As observed for recombinant strains of FMD virus (20, 21), emergent recombinants in ASF viruses may represent “game-changer” variants that complicate disease management strategies and potentially hinder the efficacy of current molecular diagnostics and vaccine candidates (18). There is growing concern that traditional veterinary protocols are no longer sufficient to address these increasingly complex biological scenarios; furthermore, the lack of definitive epidemiological research on these novel strains has exacerbated uncertainty within the global animal health community (22).

Given the rapid geographic spread of recombinant strains, synthesizing available data is urgent to support evidence-based surveillance. This study employs a scoping review methodology to evaluate both peer-reviewed and gray literature from key international bodies, such as FAO and the Global African Swine Fever Research Alliance (GARA) alongside government reports. The primary aim is to map the specific characteristics of recombinant ASFV (rASFV) genotype I/II. By identifying critical knowledge gaps and assessing the impact on traditional control measures, this review seeks to provide a robust scientific foundation to support evidence-based decision-making within regional veterinary services.

## Literature review

Following the PRISMA-ScR (Preferred Reporting Items for Systematic reviews and Meta-Analyses extension for Scoping Reviews) guideline, a systematic literature search was performed across four electronic databases—PubMed, Scopus, Web of Science and CABI—to identify relevant studies published between January 2021 and December 2025. The search strategy employed a combination of Medical Subject Headings (MeSH) and free-text keywords organized into three thematic pillars: (1) Target Virus (e.g., “African Swine Fever,” “ASFV”); (2) Genomic Characterization (e.g., “recombinant,” “inter-genotypic,” “genotype I/II,” “mosaic”); (3) Geographic Scope (e.g., “Asia–Pacific,” and specific country names).

Boolean operators (AND, OR) were utilized to intersect these themes, while the ‘NOT' operator was applied to exclude secondary literature (e.g., reviews) and studies focusing exclusively on synthetic vaccine construction or structural protein modeling. The scope was restricted to peer-reviewed original research published in English. All identified records were exported to Zotero for de-duplication and subsequent screening. To supplement the primary search, a gray literature review was conducted in both Chinese and Vietnamese. This search covered the period from January 2021 to December 2025 to identify additional records relevant to the scoping review objectives. Following the initial search, all identified records were exported to Zotero for bibliographic management and then uploaded to Rayyan (Rayyan Systems Inc., Cambridge, MA) for study selection and screening. An initial de-duplication process was performed to ensure a unique set of records. Titles and abstracts were screened against the inclusion criteria, followed by a full-text review of eligible articles. Data were then extracted using a standardized template to ensure consistency. Evidence quality was evaluated based on sequencing approach (whole-genome sequencing vs. single-locus genotyping), GenBank verification, and the availability of supporting clinical or experimental data.

## Geographic dissemination

The literature and gray literature selection process is summarized in Supplementary Figure1. Following a full-text review, nine papers met the predefined inclusion and exclusion criteria for this study.

**Figure 1:**
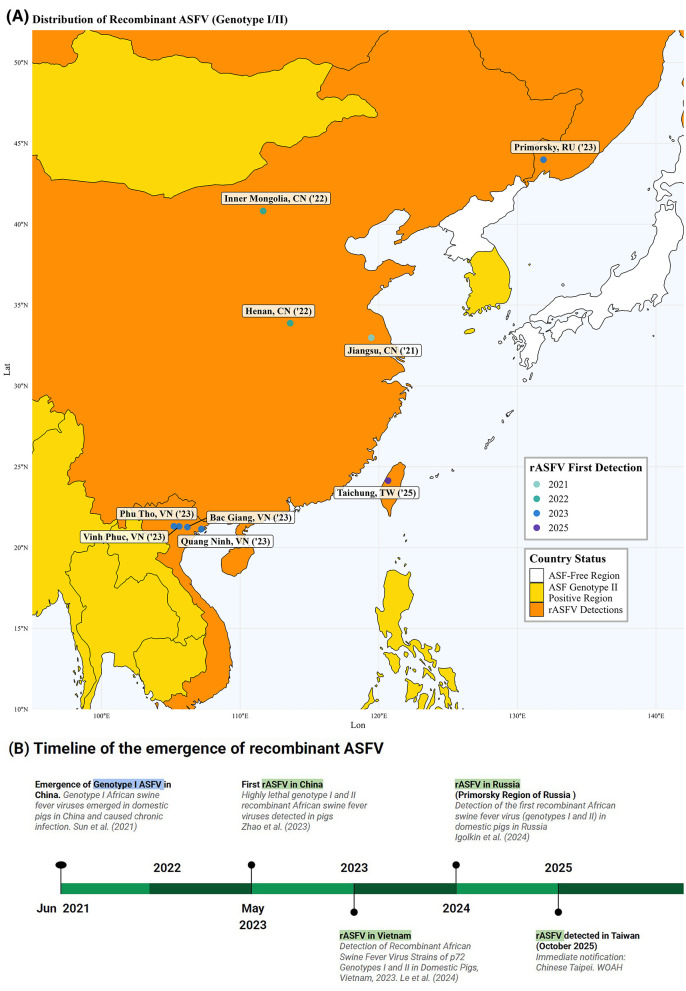
**(A)** Spatio-temporal distribution of recombinant ASFV (genotype I/II) in Asia (2021–2025). Background shading indicates ASF genotype II positive regions (since 2019), with yellow and orange highlighting countries reporting recombinant strains (rASFV). Points mark first detections: China (2021–2022: JS/LG/21, HeN/123014/22, IM/DQDM/22), Russia (May 2023), Vietnam (Sep–Oct 2023), and Taiwan (Oct 2025), illustrating the virus's transboundary expansion across affected nations. **(B)** Timeline of the emergence and geographical dispersal of recombinant ASF strains.

Since 2021, the detection of recombinant genotype I/II ASFV strains (rASFV) has followed a geographical and chronological progression in the Asia–Pacific (Figure 1). The first identifications occurred in China, beginning with Jiangsu province in 2021 (Strain: JS/LG/21), followed by detections in Henan (HeN/123014/22) and Inner Mongolia (IM/DQDM/22) throughout 2022. In a cross-sectional epidemiological study conducted in southern China in 2023, 1,275 clinical samples were tested for ASFV. The overall ASFV positivity rate was 9.25%. Among the positive cases, the detection rates of genotype I, genotype II, and co-infection with genotypes I and II were 5.02%, 3.22%, and 1.02%, respectively, indicating the presence of co-infection risk in pigs (23).

In 2023, rASFV strains were identified in domestic pigs across 11 northern provinces in Vietnam, including Hai Duong, Bac Giang, Hanoi, Phu Tho, Tuyen Quang, Thai Nguyen, Vinh Phuc, Thai Binh, Quang Ninh, Ha Giang, and Cao Bang (22, 24). Surveillance by the Vietnam Department of Animal Health and Production (DAHP) detected rASFV in 46/128 (36%) samples collected in 23 provinces in 2024; and in 336/407 (83%) samples from 34 provinces in 2025 (25, 26). Unfortunately, the current licensed ASF vaccines in Vietnam are unlikely to protect pigs against the highly virulent recombinant ASFV genotype I/II, posing a major challenge to the country's ASF control efforts (22, 25, 27). In Vietnam, the rASFV is the predominant strain after 2023. Concurrent with the expansion in Vietnam, Russia reported the presence of the virus in the Primorsky Region in May 2023.

In December 2023, Taiwan's Veterinary Research Institute reported detecting rASFV in pork products illegally brought in by travelers from China (28). Subsequently, in June 2024, the rASFV in pig carcasses that had drifted from mainland China to the sea beach of Kinmen Island was reported (29). The most recent domestic outbreak occurred in October 2025, when rASFV was identified on a farm in the Wuqi District of Taichung (30). From 2021 to 2025, the rapid spread of rASFV from China to neighboring regions marks a major epidemiological shift and underscores its growing threat to regional disease control.

## Genomic architecture of rASFV

The emergence of rASFV in the Asia–Pacific region marks a significant shift in the molecular evolution of this panzootic, characterized by a highly stable chimeric architecture. Initial genomic descriptions from China identify a genotype I backbone—specifically involving the *B646L* (p72), *E183L* (p54), and *CP204L* (p30) genes—interspersed with genotype II functional insertions (22, 31). These specific G-II insertions, most notably the *EP402R* (CD2v) and the Multigene Family (*MGF_505/360*) regions, are the primary drivers of the virus's highly virulent phenotype and its hemadsorption (HAD) activity (31, 32).

Current data point toward a monophyletic evolutionary genome profile for these recombinants. Zhao et al. (31) characterized the three index Chinese rASFV strains—JS/LG/21, HeN/123014/22, and IM/DQDM/22—which share 99.97%−99.99% homology. A comparison between the Chinese and Vietnamese rASFV strains shows that both genome profiles are categorized within the IGR-II group based on their I73R-I329L intergenic regions (24). This high degree of similarity is consistent with emergence from a common localized event rather than through multiple independent recombination incidents (34, 35). However, it may also reflect selective pressures favoring a specific recombinant architecture or a potential sampling bias. Despite this high monophyly, minor variations such as single nucleotide polymorphisms (SNPs) and insertion/deletions exists among regional detections. For instance, unique SNPs in C962R may indicate localized diversification (24), serving as a genetic marker to distinguish Vietnamese from Chinese rASFV. The overall monophyletic profile is further supported by subsequent detections in Vietnam, the Russian Primorsky region, and Taiwan, where rASFV isolates have maintained >99.9% nucleotide identity. Such a genomic evolutionary profile indicates the emergence of a transboundary lineage that has undergone rapid clonal expansion across the Asia–Pacific region, maintaining high nucleotide-level similarity between 2023 and 2025.

## Pathogenesis and clinical impact/virulence

The clinical presentation of rASFV is characterized by hybrid pathogenicity (chimeric virulence profile), as the genotype I (G-I) genetic backbone integrates high-pathogenicity markers derived from genotype II (G-II). Controlled animal challenge models in Vietnam have demonstrated that these recombinant strains exhibit a highly virulent phenotype, with mortality rates and clinical progression indistinguishable from the acute/and subacute form of the original G-II (Georgia07-like) strains (33–35). Infected animals typically experience rapid disease progression, characterized by persistent high fever and depression, anorexia, and severe systemic hemorrhage. A defining diagnostic feature of these recombinants is the acquisition of the G-II *EP402R* (CD2v) gene, which results in a consistent hemadsorption-positive (HAD+) phenotype. As a critical virulence factor typically absent in many G-I lineages, the presence of HAD+ activity serves as a vital laboratory marker for differentiating emerging recombinants from low-virulence genotype I.

## Immune evasion and vaccine ineffectiveness

A primary concern for regional disease control is the inability of genotype II (G-II) vaccines to protect against recombinant ASFV. Evidence indicates that commercial live-attenuated G-II vaccines provide negligible protection against these I/II recombinant strains (22, 31), which appear resistant to other current vaccine candidates. This lack of cross-protection is likely due to the G-I backbone of the recombinant ASFV.

## Diagnostic challenges and misclassification

The chimeric nature of rASFV introduces a potential risk of diagnostic misclassification, primarily due to a widespread G-II primer bias. Given the ASF outbreak between 2019 and 2022, many national surveillance programs in Asia–Pacific utilize PCR assays specifically optimized for the 2007 G-II lineage. This was logic and cost-effective given the epidemiological scenario; however, it is particularly problematic now that the changing epidemiological landscape—most notably in China—confirms the circulation of genotype I variants (23, 32). Consequently, these standard assays may fail to detect the G-I p72 locus entirely or, perhaps more dangerously, misidentify highly virulent recombinants as “mild” endemic G-I strains. Such a limitation in diagnostics could impact situational awareness, potentially delaying the emergency responses necessary to contain an outbreak. While multiplex real-time PCR assays capable of differentiating between ASFV G-I, G-II, and recombinant strains are increasingly used in research and available as commercial products (36–38), their integration into routine surveillance tools remains inconsistent across the region.

In China, infections with ASFV G-I are generally associated with a more chronic disease course, with clinical signs including weight loss, intermittent fever, and arthritis (19), which differ from the typically acute presentation of G-II infections. However, recently reported rASFV strains have often been detected during high-mortality outbreaks. In these cases, the clinical presentation can closely resemble that of acute G-II infection, which may lead authorities to assume a conventional G-II outbreak and delay the genomic sequencing required to identify recombination events. Continuous surveillance and monitoring of rASFV evolution are therefore essential to determine whether changes in virulence may occur.

While multiplex PCR targeting p72 and CD2v offers rapid screening, reliance on single-locus genotyping remains a weakness (39). This is compounded by sampling bias. Surveillance protocol typically prioritizes spleen and whole blood as primary target tissues (40), while swabs are considered secondary sampling options. In endemic settings in the Asia–Pacific, routine surveillance often relies on nasal or rectal swabs due to their practicality and less invasive for large-scale implementation. However, swab samples are less sensitive than lymphoid tissues or blood samples (41). For rASFV strains, evidence remains insufficient to identify sample types that optimally balance diagnostic sensitivity and operational feasibility at the national level. Further research is needed to establish evidence-based sampling strategies for effective rASF surveillance.

## Wild boar: a surveillance blind spot

While relatively uncommon, ASFV genotype II has been detected in wild boar across multiple countries in the Asia–Pacific region. Surveillance for wild boar is routine in the Republic of Korea (42), and detections have also been reported in Singapore (43), Indonesia (44), Malaysia (45), Bhutan (46), and Sri Lanka (47); risk also persists across the Southeast Asian borders of Vietnam, Laos, and Cambodia (48). Given the limitations and resources of active surveillance, underreporting is likely widespread across the region (49).

A surveillance gap exists regarding recombinant ASFV in wild boar. Only one research from the Russian Far East provides integrated data on wild boar and domestic pigs confirming rASFV in domestic swine (34, 35), surveillance in China and Vietnam remains almost exclusively focused on domestic pig farms. This neglect of the sylvatic cycle is particularly concerning given the overlapping habitats of wild boar and domestic swine in these regions.

The absence of consistent monitoring limits our understanding of the sylvatic cycle's role in the transboundary persistence and continued evolution of recombinant strains. This gap is compounded by limited research on ticks and their relationship with ASFV genotype II. The recent emergence of genotype I, genotype I/II recombinant, and gene-deleted genotype II strains in the Asia–Pacific region further complicates efforts to determine the potential roles of ticks and wild boar in ASF transmission cycles. This uncertainty contrasts with genotype I viruses in other regions, where transmission has been clearly associated with *Ornithodoros erraticus* ticks (50). Without addressing the transmission cycles blind spot, containment strategies risk overlooking reservoirs that sustain viral persistence, evolution, and future spillovers.

## Diagnostics: limitations of p72 genotyping/the demand for whole genome sequencing (WGS)

Recent genomic re-evaluations suggest that the traditional p72-based (*B646L* gene) classification into 24 genotypes may lack the methodological rigor, given the large size and complexity of ASFV genome, which this locus represents less than 1% of the viral genome (39). As p72 remains relatively stable even during significant recombination events, it is no longer a sufficient proxy for viral behavior or origin. This shift in the taxonomic paradigm further justifies the urgent transition toward whole genome sequencing (WGS) and multi-locus diagnostic assays.

Although circulating rASFV JS/LG/21-like lineage exhibits remarkably high identity (>99.9%), allowing it to traverse international borders while maintaining a stable chimeric genome. While it remains unclear if the virus has reached an evolutionary stability, subtle genomic variations suggest that micro-evolutionary drift is occurring as it moves across diverse ecological landscapes (22, 51–53). Consequently, routine WGS provides the high-resolution data necessary to track rASFV evolutionary drift and identify territory-specific mutations. This approach enhances spatiotemporal resolution, distinguishing between novel introductions and links to previous epicenters, while enabling critical virulence correlations by linking specific genomic insertions, such as those within the MGF, to clinical outcomes. For example, Hong Kong SAR successfully used high-throughput sequencing to identify ASF recombinant in November 2023 (54).

Finally, WGS serves as a vital diagnostic tool by identifying mutations in primer-binding sites; this prevents diagnostic escape and ensures that detection protocols remain effective (19, 22). By leveraging WGS data for comparison with global repositories like GenBank, the veterinary community can move toward a more granular molecular epidemiology, ultimately informing the development of next-generation, recombinant-specific diagnostics and prevention strategies.

## Underestimation of rASFV geographic detection

The inherent constraint of this scoping review is the temporal/reporting lag between initial field rASFV detection and the dissemination of peer-reviewed publication, which naturally results in an underestimation of the current geographic footprint of rASFV. While this study synthesized evidence from scientific literature and official transboundary reports from international bodies (e.g., FAO, WOAH), significant regional variations in surveillance capacity and diagnostic transparency persist across the Asia–Pacific. In several Southeast Asian countries—specifically Lao PDR, Cambodia, Thailand, Indonesia, Myanmar, and the Philippines—pronounced heterogeneity in swine production and resource constraints in molecular diagnostic infrastructure may lead to the systematic underreporting of recombinant strains. While point-of-care tests (POCTs) have become increasingly accessible in field contexts, they are generally designed for rapid “positive/negative” detection of the genotype II virus, as endemic situation. The laboratory requirements to clearly differentiate a recombinant strain from a wild-type genotype II strain necessitate a level of genomic characterization that incurs substantial extra costs and labor. For national decision-makers and veterinary services, the marginal utility of such specialized diagnostics must often be weighed against immediate outbreak containment costs, which potentially deprioritizes the identification of novel recombinants.

Furthermore, the hypothesized circulation of unauthorized or illegal live-attenuated vaccines (LAVs) significantly complicates the regional epidemiological landscape. Such practices not only serve as a primary driver for viral recombination but also create a significant disincentive for formal reporting by producers fearing legal or economic repercussions. For example, in 2026, Vietnam harbors at least three main ASFV variants: p72 genotype II, gene-deleted genotype II, and rASFV genotype I/II (55). In Thailand, both p72 genotype II and gene-deleted genotype II have also been detected in non-vaccinated swine herds (56). This “hidden” vaccine-driven evolution potentially masks the true prevalence and genetic diversity of rASFV in these endemic settings. Consequently, the findings presented in this study should be interpreted as a conservative baseline of a rapidly evolving regional situation.

The pathogenesis and disease dynamics of rASFV remain poorly understood across diverse epidemiological scenarios. Current research identifies recombination hotspots within the multigene families and structure and replication genes. To better understand these mechanisms, evolutionary comparisons with other nucleocytoplasmic large DNA viruses (NCLDV) (57), such as poxviruses, which share similar large, linear double-stranded DNA structures, may provide insights into viral dynamics. To address research gaps in rASFV, expanded genomic characterization, continued national surveillance, and strengthened regional collaboration are essential to track and mitigate the spread of these evolving strains.

## Data Availability

The datasets presented in this study can be found in online repositories. The names of the repository/repositories and accession number(s) can be found in the article/Supplementary material.
