# Supplementary material for: The evolving landscape of African swine fever: the game-changing impact of recombinant genotype I/II on Asia–Pacific control strategies
Source: Front Vet Sci. 2026 Jul 15;13:1855026. doi: 10.3389/fvets.2026.1855026 (PMC13417823; doi:10.3389/fvets.2026.1855026)
Supplement: Supplementary file 1 [file Data_Sheet_1.pdf]

Methodology: Search Strategy and Eligibility Criteria

To ensure a systematic and transparent literature review, we utilized the **Population, Intervention, Comparison, and Outcome (PICO)** framework to formulate our research question and define the eligibility criteria for study inclusion.

Element	Definition	Application in This Study
**P (Population/Problem)**	The group or population of interest.	Domestic and wild suids (*e.g., Sus scrofa domesticus*, wild boar) within the Asia-Pacific region affected by or at risk of African Swine Fever (ASF).
**I (Intervention)**	The specific exposure or event of interest.	Studies reporting the detection of recombinant ASFV (rASFV) between January 2021 and December 2025.
**C (Comparison)**	The reference point or alternative scenarios.	Comparison of different epidemiological scenarios across the Asia-Pacific region; studies comparing diagnostic performance or reporting the integration of genomic data for rASFV identification.
**O (Outcome)**	The measured results or confirmed findings.	Documentation or reports confirming rASFV through specified diagnostic methods and/or genomic sequencing.

Selection Process

The identification, screening, and inclusion of studies were conducted in accordance with the Preferred Reporting Items for Systematic reviews and Meta-Analyses extension for Scoping Reviews (PRISMA-ScR).

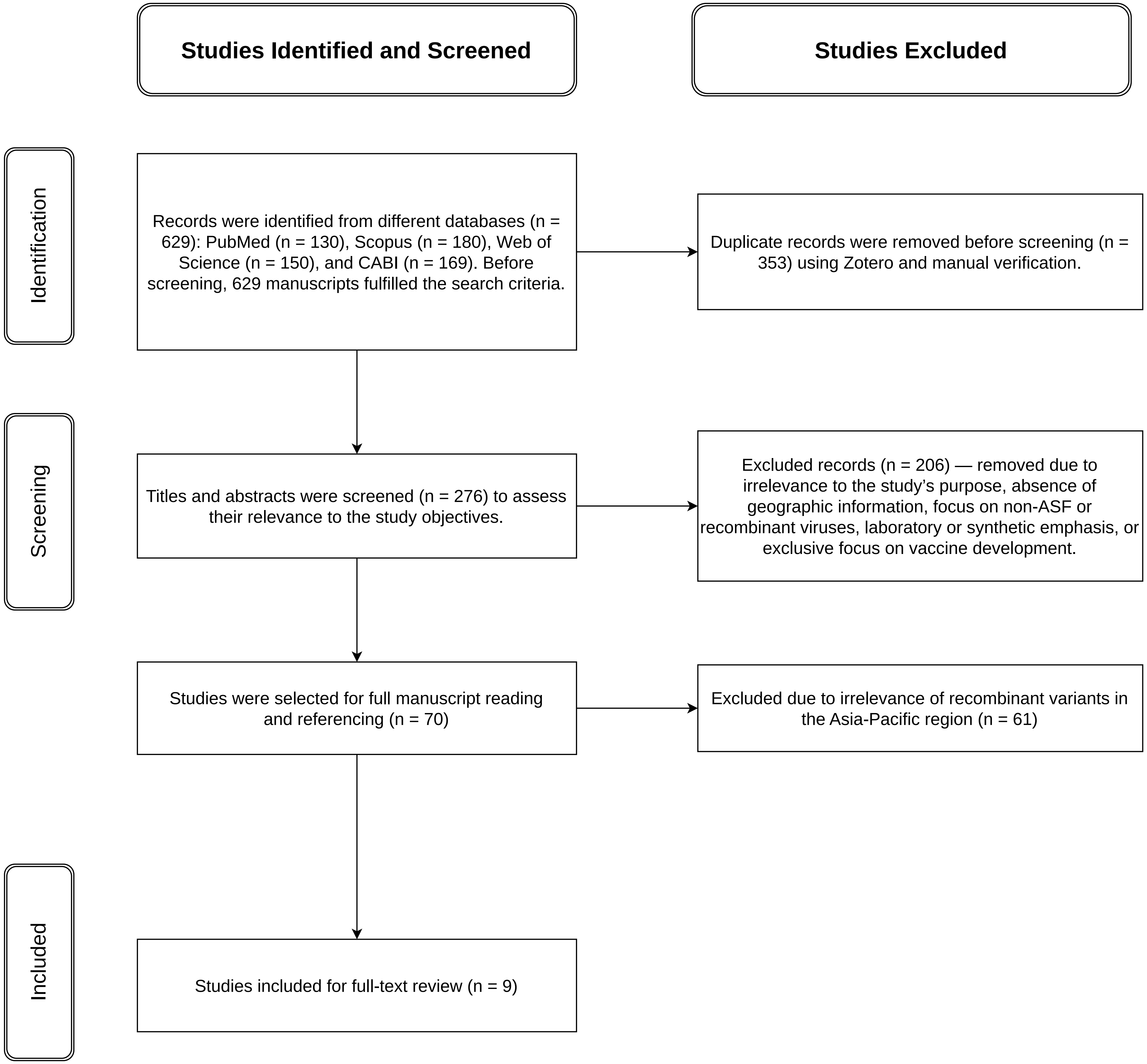
Studies Identified and Screened
Studies Excluded
Identification
Records were identified from different databases (n = 629): PubMed (n = 130), Scopus (n = 180), Web of Science (n = 150), and CABI (n = 169). Before screening, 629 manuscripts fulfilled the search criteria.
Duplicate records were removed before screening (n = 353) using Zotero and manual verification.
Screening
Titles and abstracts were screened (n = 276) to assess their relevance to the study objectives.
Excluded records (n = 206) — removed due to irrelevance to the study's purpose, absence of geographic information, focus on non-ASF or recombinant viruses, laboratory or synthetic emphasis, or exclusive focus on vaccine development.
Studies were selected for full manuscript reading and referencing (n = 70)
Excluded due to irrelevance of recombinant variants in the Asia-Pacific region (n = 61)
Included
Studies included for full-text review (n = 9)
